# Indirect Reconstruction of Pore Morphology for Parametric Computational Characterization of Unidirectional Porous Iron

**DOI:** 10.3390/ma11020193

**Published:** 2018-01-26

**Authors:** Aljaž Kovačič, Matej Borovinšek, Matej Vesenjak, Zoran Ren

**Affiliations:** Faculty of Mechanical Engineering, University of Maribor, 2000 Maribor, Slovenia; aljaz.kovacic@um.si (A.K.); matej.borovinsek@um.si (M.B.); zoran.ren@um.si (Z.R.)

**Keywords:** porous iron with unidirectional pores, compressive elastic properties, computational simulations, finite element analysis

## Abstract

This paper addresses the problem of reconstructing realistic, irregular pore geometries of lotus-type porous iron for computer models that allow for simple porosity and pore size variation in computational characterization of their mechanical properties. The presented methodology uses image-recognition algorithms for the statistical analysis of pore morphology in real material specimens, from which a unique fingerprint of pore morphology at a certain porosity level is derived. The representative morphology parameter is introduced and used for the indirect reconstruction of realistic and statistically representative pore morphologies, which can be used for the generation of computational models with an arbitrary porosity. Such models were subjected to parametric computer simulations to characterize the dependence of engineering elastic modulus on the porosity of lotus-type porous iron. The computational results are in excellent agreement with experimental observations, which confirms the suitability of the presented methodology of indirect pore geometry reconstruction for computational simulations of similar porous materials.

## 1. Introduction

**Figure 1:**
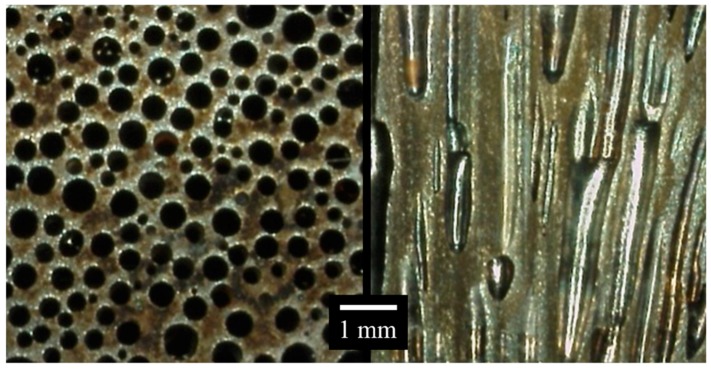
Transversal and longitudinal cross-sections of lotus-type porous iron with 44% porosity [12].

Porous materials with unidirectional pores are a unique group of materials that contain elongated cylindrical pores of finite (e.g., lotus-type or GASAR (gas armirovat, Russian for “gas reinforced”) materials [1,2]) or infinite length (e.g., UniPore structures [3,4]) aligned in the same longitudinal direction [5]. They exhibit characteristic, highly orthotropic mechanical and thermal properties due to unidirectional pore orientation, and are suitable for use in lightweight structural designs, sports equipment, as medical implants, filters, heat exchangers, etc. The continuous casting and zone melting techniques enable the fabrication of lotus-type structures (Figure 1) from a wide variety of base materials: copper, iron, steel, silver, nickel, magnesium, silicon, and even ceramics [1]. The porosity range of fabricated specimens is between 0–60%, with pore sizes varying between a few micrometres and a few millimetres. Such base material and porosity variety contributes to a very large spectrum of achievable mechanical properties. Their properties thus need to be thoroughly characterised before these materials can be used in practical engineering applications.

This study focuses on the characterisation of the base mechanical properties of lotus-type porous iron. Only a very limited number of experimental studies have been conducted so far in this regard [1,6,7,8,9,10,11]. These are crucial for the validation of any computational models, since computer simulations allow for more cost-effective virtual experimentation and also permit easier parametrical studies to estimate the behaviour of new materials.

Early computational models of lotus-type materials were rather simplistic and adequate only for coarse observations of deformation mechanisms [13,14]. The newer models follow two different approaches to the reconstruction of irregular pore geometry: (i) direct and (ii) indirect. The directly reconstructed pore geometries are based on using micro-computed tomography (µ-CT) or similar geometry acquisition techniques in combination with digital image correlation techniques [15,16,17,18,19,20]. As direct copies of real material specimens [12], they inherit realistic pore distribution and pore size. This results in the highest representativeness of computational models, but comes at a cost of having to use the µ-CT scanning and digital image correlation for every single analysed cross-section, thus losing the ability for the virtual variation of geometrical parameters (e.g., porosity). The indirect reconstruction involves approximation of realistic pore geometry, which inherently allows for certain variation of geometrical parameters. Thus far, the indirect reconstruction of lotus-type material has been limited to simplified pore geometries, comprising regular assemblies of representative volume elements (RVEs) [12,21]. They enabled effortless porosity variation through a choice of different pore sizes, but were much less representative compared to the directly reconstructed models. Also, other studies confirmed that the pore volume fraction alone is not a sufficient descriptor of a porous material’s mechanical behaviour [22,23,24,25], and must be complemented with other statistically representative geometrical parameters, for example [26,27]. The accurate computational mechanical characterisation of lotus-type material was until now only possible with direct reconstruction and modelling of the real material structure. However, Wan et al. [28] determined that pore locations in large lotus-type specimen cross-sections can be assumed as randomly spatially distributed, while Kujime et al. [15] showed that modelling lotus-type geometry with a normal distribution of pore size does not yield good computational estimations of the mechanical properties. Earlier studies in the field of fibre-reinforced composites presented numerous approaches to the automatic generation of the statistically representative distribution of circular inclusions [29,30,31,32], and it is thus feasible to expect that similar approaches can also be used to generate representative geometries of unidirectionally porous material.

The motive for this study was to develop a novel computational approach for material characterisation, using indirectly reconstructed models of any porosity with a representative pore morphology derived from the morphological analysis of a limited set of real material specimens. The geometrical representative morphology (RM) parameter is introduced to describe the unique fingerprint of planar porous structure variation at a certain porosity. The RM parameter is derived from the statistical analysis of pore morphology in real material specimens. The presented methodology was used for the parametric computational study of lotus-type porous iron to determine the dependence of engineering elastic modulus on porosity, combined with the proper validation of computational results in comparison with the available experimental data. The study was focused entirely on determining the mechanical response in the transversal direction to pore orientation, since previous research has shown that unidirectional elongated pores cause an almost linear dependency of mechanical properties on porosity [8] and negligible dependency on cross-sectional pore topology in the pore direction [12].

## 2. Methods and Computational Models

**Figure 2:**
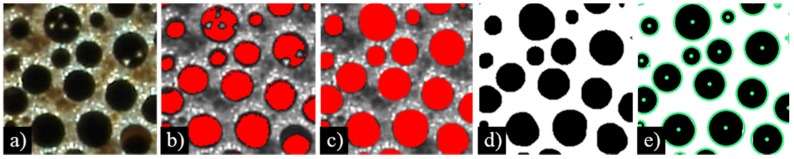
The acquisition of the specimen’s cross-section morphology data: (**a**) imported colour cross-section image. (**b**) Grayscale conversion and colour threshold. (**c**) Adjustment of image and grayscale threshold. (**d**) Conversion to black and white image and (**e**) pore recognition [12].

The procedure to define the computational models with realistic pore geometry was divided into three stages: (i) the acquisition of the specimen’s cross-sectional pore morphology data; (ii) statistical analysis of the acquired data for the derivation of representative pore morphology; and (iii) the indirect reconstruction of computer models with representative pore morphology. In the first stage, digital photographs were taken of the lotus-type iron specimen’s cross-sections oriented transversally to pore direction, as shown in Figure 2a. The digital images were then analysed with an in-house digital image correlation code for automatic image recognition and data acquisition from imported digital photographs. The code algorithm analysed colour differences between pores and the base material. It isolated all pixels with colour values above a certain threshold (pore candidates are highlighted red in Figure 2b), and identified pixel clusters (pores) by making lists of pore candidates that shared common neighbours. The algorithm relied on human supervision with a global threshold adjustment that excludes all regions of base material from pore candidates (Figure 2b), and local touch-up threshold adjustment, which defines more accurate and smooth pore boundaries (Figure 2c). Figure 2d shows an example of recognised pore geometry used for data acquisition. The number of pixels in each pixel cluster was associated with its area; whereas the arithmetic mean position of the pixel centres was associated with its centroid location. The pore pixel cluster area *A* was converted to pore diameter $D=2\cdot \sqrt{A/\pi }$ by assuming pore approximation with a perfect circle at determined centroid locations, as shown in Figure 2e.

For easier comparison between the data acquired from different specimen images, the pore diameters were normalised with the average pore size in the analysed cross-section. The normalised pore diameters were then classified into seven different size classes, ranging from the minimum to the maximum computed values (0–2.1). The percentage of pores in each size class was calculated by dividing the number of pores in each size class with the total number of pores in the analysed cross-section. These pore percentages were then defined as the representative morphology (RM) parameters, a unique fingerprint of the observed specimen’s cross-section with a certain porosity, following a similar methodology as described in [28]. It was discovered that this simplified descriptor is sufficient to assure good accuracy in case of analysed lotus-type porous iron by using the developed methodology in computational experimentation. For some porous materials with particular spatial pore pattern repeatability, it might be necessary to include also the spatial relationship of pores together with the distribution of pore sizes for more accurate indirect reconstruction modelling, following e.g., the methodology as explained in [28].

Eight different cross-sections of the lotus-type porous iron (e.g., Figure 1) were analysed in this way, two for each fabricated specimen. Two specimens had an average porosity of 22% and two an average porosity of 43%. For porosities ranging between 22% and 43%, the RMs were estimated through simple linear interpolations of pore percentages for each size class. Similarly, the linear extrapolations were used for the evaluation of the RMs at porosities lower than 22% and higher than 43%. If the extrapolated pore percentage of a certain pore size class reached a negative value, it was corrected to 0%, and all the percentages were scaled again to the sum of 100%.

An algorithm for the indirect reconstruction of representative computer models with arbitrary porosity was developed to generate a finite set of pores with a certain porosity value. The model control parameters were the porosity, the average pore diameter and the model size. The model pore set was generated in the following way: for each new pore, a random function, weighted with the RM, was used to select a pore size class, from which an un-weighted random function selected the final pore size. This process was repeated until the chosen porosity was reached in the defined model size. The pores were assumed to be randomly distributed over the lotus-type material cross-section according to Wan et al. [28]. Therefore, all created pores were sorted by their size and sequentially randomly positioned in the dedicated model area, starting with the largest and ending with the smallest pore. As no pore overlapping was observed in real specimen analysis, the minimum possible distance between pores was assigned, as observed from a digital image correlation analysis of real specimen cross-sections. However, the pores were allowed to overlap with the boundaries of the designated model area.

The total of 20 models with different porosities, gradually ranging from 0% to 72% were reconstructed. The percentage of large pores had to be slightly reduced in favour of a small pore percentage for porosities higher than 65%, since the simple linear extrapolation of pore percentages failed to satisfy the non-overlapping criterion of the pores. Twenty indirectly reconstructed models (one for every porosity value) were used for the computational characterisation of the engineering elastic modulus of lotus-type porous iron in the transversal direction. The finite element method (FEM) software system Abaqus/Standard [33] was used for computational simulations following the approach presented in [12]. Each indirectly reconstructed cross-section was square-shaped and extruded into a 3D geometry along pore direction for 1/4 of its height. Symmetrical boundary conditions were prescribed in three orthogonal directions to virtually increase the size of analysed representative volume element (RVE) by eight times. The compressive, displacement driven loading in transversal direction was prescribed until 2% of global engineering strain. Only elastic behaviour of the base material (iron) was taken into account, with a Young’s modulus of 210 GPa and Poisson’s ratio 0.3. The geometry was meshed with a hexahedra-dominated mesh, consisting of quadratic hexahedral 3D-stress elements with reduced integration (type C3D20R) and quadratic wedge elements (type C3D15). The number of elements ranged between 25,000–75,000, depending on the model’s porosity and geometry. The observed simulation results were reaction loads, which were normalised over the RVE area to calculate the engineering stress. The resulting linear engineering stress–strain relationship was used to determine the effective elastic modulus for each porosity. The computed elastic moduli of lotus-type iron were compared with experimentally determined values from acoustic measurements by Tane et al. [8]. Experimentally characterised specimens were fabricated from the same batch as the ones analysed in this study.

**Figure 3:**
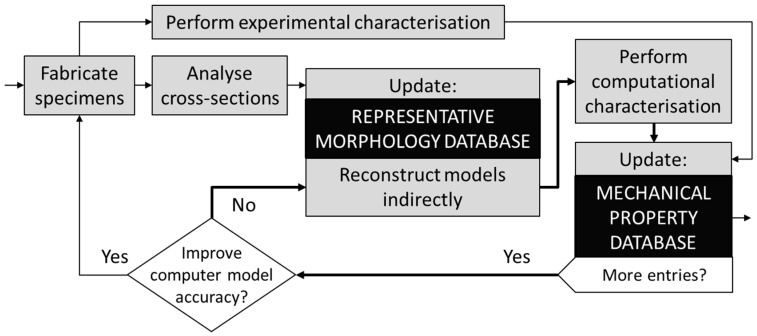
The algorithm for the computational characterisation of mechanical properties with use of indirectly reconstructed computer models.

Figure 3 presents an algorithm for the mechanical characterisation of lotus-type porous material, using indirectly reconstructed computer models with a representative morphology. It is important to note that numerous specimens with similar porosities have to be statistically analysed for sufficient accuracy of the RM database. The statistical analysis has to be made for more than one porosity to allow for correct interpolation and extrapolation of RMs, which in turn can lead to higher-order approximations as opposed to the only linear approximations used in this first study. It is also suggested to investigate the fabricated specimens experimentally once they are statistically analysed, and update the mechanical property database. When the RM database has enough entries (eight in our case of porous iron) and the indirectly reconstructed computer models are validated, the experimental data acquisition can be omitted. The computer models of any porosity value can then be indirectly reconstructed from the database for use in computational simulations. Computationally evaluated properties can then be imported into the mechanical property database. This procedure is represented with the bold line in Figure 3, and can be repeated numerous times to complement the mechanical property database.

## 3. Computational Results

**Figure 4:**
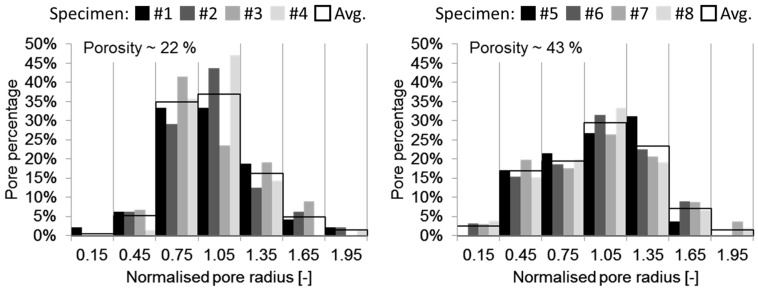
The results of the cross-section pore analysis for lotus-type iron.

The results of the geometrical analysis of eight cross-sections of the lotus-type porous iron are shown in Figure 4. The graphs show the percentages of pores in different classes of normalized pore radius size. The corresponding numerical values are given in Table 1. The total numbers of analysed pores in specimens with 22% and 43% porosities are 256 and 502 respectively, which is a sufficient sample size for the statistical representativeness of the results.

Pore geometry analysis of 22% average porosity lotus-type iron shows a very small percentage of small and large pores. Most of the pores are of an average normalised pore radius 1. The RMs of specimens at 22% average porosity roughly follow a normal distribution, with the exception of the third specimen. The largest differences in pore percentages can be observed in the class with an average normalised pore size of 1.05, where scattering reaches almost 50%. Specimens with 43% average porosity also have a majority of pores with an average normalised pore radius of 1, but the size concentration is more intensive than in 22% average porosity specimens. The scattering of pore percentages between different specimens is also smaller for 43% average porosity, with only 39% in the pore size class of 1.35. The highest percentage of pores is 33% in the 1.05 pore size class, and the lowest 0% in the 0.15 and 1.95 size classes. The largest value of average pore percentage at 22% average porosity is 37% in the 1.05 size class, and the lowest is less than 1% in the 0.15 size class. For 43% average porosity, the highest averaged pore percentage reaches 30% in the 1.05 size class, and the lowest lies between 1% and 2% in the 1.95 size class.

**Figure 5:**
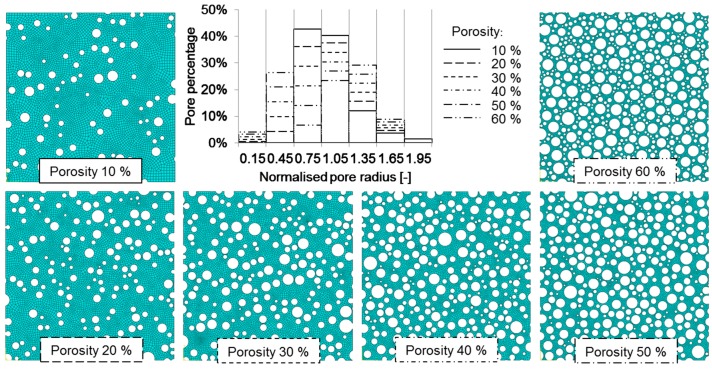
The indirectly reconstructed lotus-type cross-sections with different porosities and their corresponding pore percentages.

Figure 5 shows the graph with interpolated and extrapolated values of RMs for different chosen porosities and corresponding images of indirectly reconstructed models. The corresponding RM values are listed in Table 2. The six different porosities always follow the same order due to linear interpolation and extrapolation of the pore percentage. However, the direction of this order varies, as the pore percentages in pore size classes of 0.15, 0.45, 1.35 and 1.65 grow with increasing porosity, and percentages in pore size classes of 0.75 and 1.05 decline with increasing porosity. The change of pore percentage in the 1.95 pore size class is too small for observation. The largest inverse dependency of pore percentage on porosity is observed in the 0.75 pore size class, where the pore percentage decreases from 42% at 10% porosity to 6% at 60% porosity. This pore percentage would reach negative values at approximately 70% porosity by following this trend. Pore percentages would also reach negative values at lower than 10% porosities in the 0.15 and 0.45 size classes. In all these cases, the values of pore percentage are restricted to the minimum of 0%, and the linear extrapolation of pore percentages is omitted. Figure 5 presents the indirectly reconstructed models for six different porosities, and allows for a simple visual comparison. Although their morphologies may look completely random at the first glance, they are statistically equivalent to those of real lotus-type iron, and exhibit a representative mechanical response, as discussed below.

**Figure 6:**
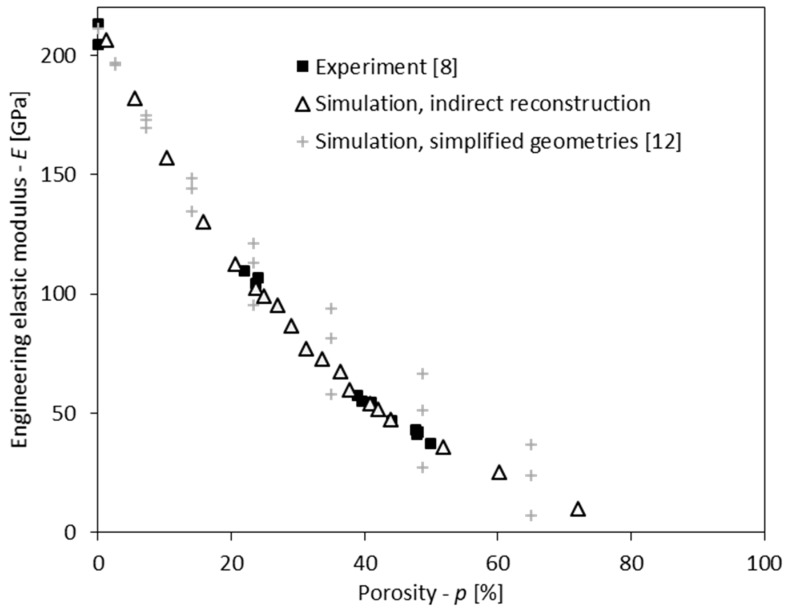
The dependency of engineering elastic modulus in transversal direction to pores on porosity in lotus-type iron: comparison of experimental measurements [8] with simulation results for indirectly reconstructed geometries and simplified geometries [12].

The graph in Figure 6 presents the effect of porosity on the engineering elastic modulus for lotus-type porous iron, obtained with experimental measurements and computer simulations. The computational results of indirectly reconstructed geometries are also presented in Table 3. The experimental results are available for porosities of 20%, 40% and 50%. For 0% porosity, the experimental results represent the elastic modulus of nonporous iron, fabricated with the same casting technique as the lotus-type porous iron. The computational results of models with simplified pore geometry are published in [12] and are included for comparison purposes only.

## 4. Discussion

The pore morphology of lotus-type iron specimens notably varies with the porosity, and pore sizes can be associated with normal distribution only for porosity values near 30%. At very low porosities (0–20%), the differences in pore sizes are smaller than at higher porosities. The majority of pores (up to 85%) are of the nominal pore size classes of 0.75 and 1.05. The diversity of pores grows with an increase in porosity, and at around 50%, a gap starts to form in the 0.75 size class. Afterwards, the majority of pores move away from the values of the average pore size. They become either small or large, while the number of averagely-sized pores decreases. At 60% porosity, it is estimated that already 70% of pores lie outside the 0.75 and 1.05 nominal pore size classes. This phenomenon can be observed in the model with 60% porosity (Figure 5), where smaller pores surround larger pores, and pores of intermediate size are rare. Although this observation is not yet confirmed in real material specimens, a lack of intermediately-sized pores at high values of porosity seems inevitable because of dense pore packing and more homogenous material distribution between them. However, the close packing and high diversity of pores at porosities higher than 60% may also be related to some contemporary challenges in the fabrication of highly porous lotus-type material. Furthermore, for porosities higher than 65%, any RM obtained with linear extrapolation is no longer appropriate for the presented reconstruction methodology, since it fails to fulfil the “no pore overlapping” criterion under the random distribution of pores over the dedicated area, even for 10^5^ insertion attempts. Consequently, the percentage of smaller pores has to be manually increased to pass the linear extrapolation values, which yields successful model reconstruction. It should be noted that the engineering elastic modulus values at porosities higher than 50% estimated by indirect computational simulations have to be further validated by the experimental testing of real specimens of such porosity, when they become available. The question thus remains of how accurately the extrapolated pore percentages would represent the morphology of realistic lotus-type materials with very high porosity, since they have yet to be fabricated with technological advances. Even though the computational results can approximately predict the expected behaviour at higher porosities, it cannot be expected that the realistic material would have the same pore morphology. The question may be answered in the future, when the fabrication techniques are improved for the production of lotus-type materials with a porosity of above 50%. On the other hand, the need for the manual adjustment of pore percentages at high porosities indicates that linear interpolation and extrapolation is not the optimal choice for the reconstruction of models with extreme values of porosity. In the range of currently achievable porosities up to 50%, the linear relation provides for sufficient accuracy, since good correlation between experimental and computational results is observed. Conveniently, the presented algorithm allows numerous updates of the RM database with potential for the improvement of computational characterisation.

The results of the analysed specimens at 22% average porosity show a greater scattering of morphology statistics than at 43% porosity. This statistical deviation might be caused by a smaller statistical sample due to the lower number of pores in the examined specimens. On the other hand, more space between the pores provides less restrictions on size and allows for easier development of statistical diversity during the fabrication process. However, the averaging statistics between similar porosities is considered to be necessary due to such deviations.

The final pore percentages and porosities of reconstructed models differ slightly from the desired values in the reconstruction process. The indirectly reconstructed pore morphologies can differ from the target RM due to chosen randomness, but the statistical scatter decreases with the size of pore set used for geometry generation. The terminal porosity can also deviate from the target value; partly due to randomness in the pore selection process, and partly because the small portion of the generated pore set that stretches beyond the model boundaries is excluded from the final porosity calculation. Both effects decrease by increasing the model size relative to the average pore size. However, the discussed discrepancies did not compromise the computational characterisation, since the exact achieved porosity value was again calculated for each model after its generation.

The computational characterisation of engineering elastic modulus with indirectly reconstructed lotus-type models is in very good agreement with the experimental results at comparable porosities, and the scatter of computational results is similar to that of the experimental results. The engineering elastic modulus was accurately estimated for the entire currently achievable porosity range of lotus-type iron. This proves the validity of indirectly reconstructed computational models and the presented methodology of indirect geometry reconstruction. Furthermore, the accuracy of models with indirectly reconstructed pore geometry is significantly better than that of models with simplified pore geometry. This confirms the importance of realistic pore geometry in the lotus-type material modelling, which is now simpler due to the validated indirect reconstruction algorithm.

## 5. Conclusions

The algorithm for the indirect reconstruction of realistic pore geometries enables accurate computational prediction of a lotus-type material’s properties. It is based on: (i) data extraction through image recognition; (ii) statistical analysis for evaluation of the representative morphology (RM) parameter; and (iii) indirect reconstruction of computer models with statistically and therefore mechanically representative pore morphology.

Statistical analysis of lotus-type porous iron’s cross-sections shows that pore morphology depends heavily on porosity, which in turn strongly influences the material’s elastic behaviour. Furthermore, the study confirms the findings of Wan et al. [28], that pores are randomly spatially distributed in large material specimens of lotus-type material.

An example of the mechanical characterisation of lotus-type porous iron over the entire range of porosity was given based on indirectly reconstructed computer models with realistic pore morphology. There was very good agreement of the computational and experimental results in terms of the elastic modulus dependence on porosity for porosities up to 50% porosity validates the suitability of the indirect reconstruction methodology for the computational characterisation of the lotus-type materials. Furthermore, the accuracy of models with indirectly reconstructed pore geometry is significantly better than that of previous models with simplified pore geometries.

The presented methodology can be used for computational analyses of other unidirectional porous structures with cylindrical pores, or for the characterisation of lotus-type porous structures of various base materials. Statistical analyses of their geometry shall reveal if the pore morphology is significantly influenced by the type of base material. In addition, the characterisation of other mechanical or thermal properties like yield stress, densification strain, hardening rate and heat conduction constants is also possible.

## Figures and Tables

**Table 1:** Porosities, numbers of pores and pore percentages in the analysed lotus-type iron specimens with 22% and 43% average porosity.

Parameters		Pore Percentage (%)					Pore Percentage (%)				
Specimen		#1	#2	#3	#4	Avg.	#5	#6	#7	#8	Avg.
Porosity (%)		20	19	20	27	22	40	45	43	44	43
No. of pores (-)		48	48	89	71	64	135	124	137	106	126
Norm. pore radius (-)	0.15	2	0	0	0	1	0	3	3	4	3
	0.45	6	6	7	1	5	17	15	20	15	17
	0.75	33	29	42	36	35	22	19	18	20	19
	1.05	33	44	24	47	37	27	32	27	33	30
	1.35	19	13	19	14	16	31	23	21	19	23
	1.65	4	6	9	0	5	4	9	9	7	7
	1.95	2	2	0	1	1	0	0	4	2	1

**Table 2:** Interpolated and extrapolated pore percentages for various porosities.

Parameters		Pore Percentage (%)						
Porosity (%)		0	10	20	30	40	50	60
Norm. pore radius (-)	0.15	0	0	0	1	2	3	4
	0.45	0	0	4	10	15	21	26
	0.75	47	43	36	29	21	14	7
	1.05	41	40	37	34	30	27	23
	1.35	8	12	16	19	22	26	29
	1.65	2	4	5	6	7	8	9
	1.95	1	1	1	1	1	1	1

**Table 3:** The engineering elastic modulus of lotus-type iron in transversal direction to pores, obtained computationally with indirect model reconstruction.

*p* (%)	*E* (GPa)	*p* (%)	*E* (GPa)	*p* (%)	*E* (GPa)	*p* (%)	*E* (GPa)
0	210	21	112	31	77	42	51
1	206	24	102	34	72	44	48
6	182	25	99	36	68	52	36
10	157	27	95	38	60	60	25
16	130	29	86	41	54	72	10

## References

[1] Nakajima H. (2007). Fabrication, properties and application of porous metals with directional pores. Prog. Mater. Sci..

[2] Shapovalov V.I. (1994). Porous Metals. MRS Bull..

[3] Vesenjak M., Hokamoto K., Anžel I., Sato A., Tsunoda R., Krstulović-Opara L., Ren Z. (2015). Influence of the explosive treatment on the mechanical properties and microstructure of copper. Mater. Des..

[4] Hokamoto K., Vesenjak M., Ren Z. (2014). Fabrication of cylindrical uni-directional porous metal with explosive compaction. Mater. Lett..

[5] Lehmhus D., Vesenjak M., Schampheleire S., Fiedler T. (2017). From Stochastic Foam to Designed Structure: Balancing Cost and Performance of Cellular Metals. Materials.

[6] Nakajima H., Tane M., Buschow K.H.J., Robert W.C., Merton C.F., Bernard I., Edward J.K., Subhash M., Patrick V. (2010). Properties of Lotus-type Porous Metals. Encyclopedia of Materials: Science and Technology.

[7] Hyun S.-K., Ikeda T., Nakajima H. (2004). Fabrication of lotus-type porous iron and its mechanical properties. Sci. Technol. Adv. Mater..

[8] Tane M., Ichitsubo T., Nakajima H., Hyun S.K., Hirao M. (2004). Elastic properties of lotus-type porous iron: Acoustic measurement and extended effective-mean-field theory. Acta Mater..

[9] Tane M., Ichitsubo T., Hyun S.K., Nakajima H. (2005). Anisotropic yield behavior of lotus-type porous iron: Measurements and micromechanical mean-field analysis. J. Mater. Res..

[10] Nakajima H., Ikeda T., Hyun S.K. (2004). Fabrication of Lotus-type Porous Metals and their Physical Properties. Adv. Eng. Mater..

[11] Nakajima H., Tane M., Hyun S.K., Seki H., Zhao H., Fleck N.A. (2009). Anisotropic Mechanical Properties of Lotus-Type Porous Metals.

[12] Vesenjak M., Kovačič A., Tane M., Borovinšek M., Nakajima H., Ren Z. (2012). Compressive properties of lotus-type porous iron. Comput. Mater. Sci..

[13] Kee A., Matic P., Popels L. (1997). A two dimensional computational study of a gasar porous copper microstructure. Mater. Sci. Eng. A.

[14] Kee A., Matic P., Everett R.K. (1998). A mesoscale computer simulation of multiaxial yield in gasar porous copper. Mater. Sci. Eng. A.

[15] Kujime T., Tane M., Hyun S.K., Nakajima H. (2007). Three-dimensional image-based modeling of lotus-type porous carbon steel and simulation of its mechanical behavior by finite element method. Mater. Sci. Eng. A.

[16] Kujime T., Nakajima H. (2010). Investigation of the Mechanical Properties of Lotus-type Porous Carbon Steel made by Continuous Zone Melting Technique. Mater. Sci. Forum.

[17] Fiedler T., Veyhl C., Belova I.V., Tane M., Nakajima H., Bernthaler T., Merkel M., Öchsner A., Murch G.E. (2011). On the Anisotropy of Lotus-Type Porous Copper. Adv. Eng. Mater..

[18] Borovinšek M., Vesenjak M., Matela J., Ren Z. (2008). Computational reconstruction of scanned aluminum foams for virtual testing. J. Serbian Soc. Comput. Mech..

[19] Sulong M.A., Vesenjak M., Belova I.V., Murch G.E., Fiedler T. (2014). Compressive properties of Advanced Pore Morphology (APM) foam elements. Mater. Sci. Eng. A.

[20] Fiedler T., Sulong M.A., Vesenjak M., Higa Y., Belova I.V., Öchsner A., Murch G.E. (2014). Determination of the thermal conductivity of periodic APM foam models. Int. J. Heat Mass Transf..

[21] Kovačič A. (2011). Karakterizacija Mehanskih Lastnosti Poroznega Gradiva z Vzdolžnimi Porami s Parametričnimi Računalniškimi Simulacijami = Characterisation of Mechanical Properties of Porous Materials with Longitudinal Pores with Parametric Computer Simulations. Undergraduate Thesis.

[22] Bilger N., Auslender F., Bornert M., Moulinec H., Zaoui A. (2007). Bounds and estimates for the effective yield surface of porous media with a uniform or a nonuniform distribution of voids. Eur. J. Mech. A/Solids.

[23] Bilger N., Auslender F., Bornert M., Michel J.-C., Moulinec H., Suquet P., Zaoui A. (2005). Effect of a nonuniform distribution of voids on the plastic response of voided materials: A computational and statistical analysis. Int. J. Solids Struct..

[24] Sevostianov I., Kushch V. (2009). Effect of pore distribution on the statistics of peak stress and overall properties of porous material. Int. J. Solids Struct..

[25] Gǎrǎjeu M., Suquet P. (2007). On the influence of local fluctuations in volume fraction of constituents on the effective properties of nonlinear composites. Application to porous materials. J. Mech. Phys. Solids.

[26] Torquato S. (1995). Nearest-neighbor statistics for packings of hard spheres and disks. Phys. Rev. E.

[27] Romanov V., Lomov S.V., Swolfs Y., Orlova S., Gorbatikh L., Verpoest I. (2013). Statistical analysis of real and simulated fibre arrangements in unidirectional composites. Compos. Sci. Technol..

[28] Wan J., Li Y., Liu Y. (2007). Spatial distribution of pores in lotus-type porous metal. J. Mater. Sci..

[29] Vaughan T.J., McCarthy C.T. (2010). A combined experimental-numerical approach for generating statistically equivalent fibre distributions for high strength laminated composite materials. Compos. Sci. Technol..

[30] Yang L., Yan Y., Ran Z., Liu Y. (2013). A new method for generating random fibre distributions for fibre reinforced composites. Compos. Sci. Technol..

[31] Wang Z., Wang X., Zhang J., Liang W., Zhou L. (2011). Automatic generation of random distribution of fibers in long-fiber-reinforced composites and mesomechanical simulation. Mater. Des..

[32] Melro A.R., Camanho P.P., Pinho S.T. (2008). Generation of random distribution of fibres in long-fibre reinforced composites. Compos. Sci. Technol..

[33] SIMULIA (2011). Products: Abaqus FEA.

